# PP107 Effectiveness And Safety Of Cytoreductive Surgery And Heated Intraperitoneal Chemotherapy For Pediatric Peritoneal Carcinomatosis: A Living Evidence Synthesis

**DOI:** 10.1017/S026646232400268X

**Published:** 2025-01-07

**Authors:** Nora Ibargoyen Roteta, Josune Domínguez García, Ainhoa Jausoro-Zubiaga, Lorea Galnares-Cordero, Iñaki Gutierrez-Ibarluzea, Maria X. Rojas, Ariadna Auladell-Rispau, Francisca Verdugo-Paiva

## Abstract

**Introduction:**

The evidence synthesis developed to inform decision-making on the use of cytoreductive surgery (CRS) and heated intraperitoneal chemotherapy (HIPEC) for pediatric peritoneal carcinomatosis showed that currently available evidence is of very low quality. As new evidence could arise within the following months, we adopted a rigorous living evidence synthesis (LES) approach to provide a timely update and favor decision-making based on actual evidence.

**Methods:**

This LES started with a baseline synthesis about the effects of CRS and HIPEC on pediatric peritoneal carcinomatosis. On 31 August 2023, we set up the evidence monitoring for up to 12 months. Following the Living Evidence to Inform Health Decisions (LE-IHD) framework, we planned and developed the evidence monitoring, supported by technological enablers. We searched for ongoing studies in trial registries every three months. New eligible studies were assessed following a systematic and reproducible process to decide on their incorporation in the evidence summary. This process was periodically reviewed to determine the continuation/withdrawal of the living mode.

**Results:**

The baseline synthesis identified one systematic review suggesting that CRS and HIPEC could increase overall survival in pediatric peritoneal carcinomatosis (very low-quality evidence), but no comparative data could be obtained against usual care. To date, the evidence monitoring has not identified new relevant studies on the impact of CRS and HIPEC in overall and disease-free survival, morbidity, or quality of life in pediatric peritoneal carcinomatosis. At the time of the conference, we will report on nine months of monitoring and regular updates including key messages on any changes in the evidence synthesis conclusions.

**Conclusions:**

For HTA reports based on very low-quality evidence (uncertain results), the LE approach allows for timely updating of conclusions, adding value in decision-making. The LE-IHD framework facilitates HTA developers’ tasks for planning and conducting LE synthesis to inform health decisions.

